# Relationship Between Explosive Strength Capacity of the Knee Muscles and Deceleration Performance in Female Professional Soccer Players

**DOI:** 10.3389/fphys.2021.723041

**Published:** 2021-10-11

**Authors:** Qingshan Zhang, Aurélie Léam, Alexandre Fouré, Del P. Wong, Christophe A. Hautier

**Affiliations:** ^1^Université de Lyon, UCBL-Lyon 1, Laboratoire Interuniversitaire de Biologie de la Motricité, Villeurbanne, France; ^2^School of Nursing and Health Studies, The Open University of Hong Kong, Ho Man Tin, Hong Kong, SAR China

**Keywords:** deceleration performance, peak torque, RTD, H/Q ratio, female, soccer

## Abstract

The present study aimed to investigate the relationship between linear deceleration performance and explosive strength capacity of the knee muscles. Fourteen female professional soccer players completed the maximal sprint deceleration tests and knee flexor (KF) and knee extensor (KE) isokinetic concentric (240° and 60°.s^−1^) and eccentric contractions (30°.s^−1^). Linear deceleration performance was evaluated from horizontal breaking force (*F*_H_), power (*P*_H_), and impulse (*I*_H_) during a maximal linear deceleration. The peak torque (PT) of KF and KE, PT ratio between KF and KE (conventional and functional H/Q ratio), rate of torque development (RTD) for each muscle group, and RTD between KF and KE (RTD H/Q) were extracted from the isokinetic contractions. Pearson’s correlation coefficients revealed that the eccentric (30°.s^−1^) and concentric (60°.s^−1^, 240°.s^−1^) KE peak torque, and the concentric KF peak torque (240°.s^−1^) were significantly correlated with *F_H_, P_H_*, and *I_H_* (−0.75<*r*<−0.54). Moreover, a significant correlation was found between KE RTD during eccentric contraction and *F_H_*, *P_H_*, and *I_H_* (−0.63<*r*<−0.54). Besides, a significant correlation was observed between RTD H/Q at 60°.s^−1^ and *P_H_*, *I_H_* (−0.61<*r*<−0.57). No significant relationship was observed between the H/Q ratio, KF RTD and deceleration performance. These main findings indicated the importance of the ability to quickly produce high KE eccentric torque, contributing to braking force production. Meanwhile, RTD H/Q should be assessed for its essential role in knee joint dynamic stability and can be a relevant index to determine deceleration performance.

## Introduction

In most team sports such as soccer or rugby, the ability to decelerate quickly while sprinting at high speed could allow rapid re-acceleration or change-of-direction and therefore overtaking opponents in the decisive situations (Buchheit et al., 2014; Vigh-Larsen et al., 2018). Previous external movement analysis in soccer reported that the players perform approximately 14–26 high intensity of the acceleration (≥3m.s^−2^), and the 43–56 deceleration (≤−3m.s^−2^) in short-duration less than 1s across the competitive match (Wehbe et al., 2014; Russell et al., 2015, 2016; de Hoyo et al., 2016). Also, numerous studies have suggested that one of the critical phases for change-of-direction is the rapid body deceleration before the re-acceleration phase (Dos’Santos et al., 2017; Jones et al., 2017). Yet, deceleration is employed to stop or quickly decrease the body’s center of mass velocity before a change-of-direction (Hewit et al., 2011) inducing substantial braking force followed by a propulsive force with high sprint velocity, which requires lower limb muscle qualities such as strength, power and reactive strength capacity (Brughelli et al., 2008). Moreover, deceleration combined with a change-of-direction or cutting maneuver has been identified as the movement that can induce non-contact injuries such as anterior cruciate ligament (ACL) injury (Alentorn-Geli et al., 2009) due to the high level of ACL strain induced by the substantial external knee valgus moment (McLean et al., 2004). Indeed, female soccer players are more likely to suffer ACL injury who tended to have decreased knee flexion angle and increased knee valgus angle compared to males during the critical movement (cutting, change-of-direction change-of-direction), which could induce ACL injury due to the excessive anterior shear forces (Yu et al., 2002). Although the kinematics and kinetics of running sprints and neuromuscular determinants of sprinting performance have been extensively studied in the literature, the mechanical deceleration ability has been less investigated, especially in female soccer players.

Performance in change-of-direction is influenced by numerous factors, including lower limb strength (Jones et al., 2017; Harper et al., 2021), reactive force (Castillo-Rodriguez et al., 2012), body stability (Sasaki et al., 2011). During the deceleration maneuver, the lower limb absorbs the kinetic energy by contracting the knee extensor (KE) muscles eccentrically to decrease body momentum and stop as fast as possible in a stable posture. In this regard, the KE eccentric torque may be essential to perform that kind of movement. Happer et al. (2018) indicated that KE eccentric torque at 60°.s^−1^ was largely correlated to deceleration performance (i.e., time to stop, distance to stop; Harper et al., 2021). It could be imagined that the better eccentric capacity of KE permits the athlete to absorb the higher kinetic energy to decelerate. However, ground contact occurs ahead of the center of mass during deceleration with a more extended knee and a flexed hip angle compared to the acceleration phase (Hewit et al., 2011). This movement’s organization induces large internal knee constraints due to the great braking forces requiring a fast contraction of agonist and antagonist muscles to protect the joint structures with a high knee joint stability. Therefore, a high coordination and torque balance between knee flexor (KF) and KE should be considered as a potential determinant of knee joint stability, which may assist deceleration performance. From a clinical point of view, the hamstring-to-quadriceps strength ratio (H/Q ratio) was usually used to evaluate the balance between KF and KE. Additionally, a high rate of torque development (RTD) was suggested to be determinant in sport performance and musculoskeletal injuries prevention (Rodriguez-Rosell et al., 2018; Ishoi et al., 2019). More specifically, Zebis et al. (2011) suggested that a rapid hamstring-to-quadriceps strength ratio (RTD H/Q) appears of utmost interest to assess knee joint dynamic stability during explosive movement (Zebis et al., 2011), indicating the ability to rapidly increase the level of force produced by KF and KE in order to maintain knee joint dynamic stability. Consequently, it seems that the higher H/Q ratio contributes to the better knee joint stability, permitting the athlete to maintain a stable knee joint and body position to decelerate.

However, to the best of our knowledge, previous research only examined the relationships between the peak torque (PT) of the lower limb and deceleration performance but ignored the role of explosive neuromuscular capacity to rapidly produce torque and the balance between knee flexor and knee extensor contributions with regards to maximal deceleration performance. Therefore, the aims of the study were: (i) to determine the relationships between the knee muscle isokinetic strength profile and sprint deceleration performance, and (ii) to examine the relationship between explosive neuromuscular capacity (RTD and RTD H/Q) and deceleration performance. We hypothesized that (i) knee extensor eccentric and knee flexor concentric peak torque should be correlated to deceleration performance with higher braking force and power production and (ii) the explosive torque capacities of KF and KE, and their related ratios (RTD H/Q) may also significantly impact deceleration performance whereas no relationship between the traditional H/Q ratio and deceleration performance was expected.

## Materials and Methods

### Participants

Fourteen French national-level female soccer players (Height: 166.1±5.9cm, Mass: 63.1±7.7kg, Age: 24.7±4.2years, training volume: 12.4±2.7h.week^−1^, experience training: 11.4±5.9years) volunteered to participate, with four training sessions per week. All participants had had no lower extremity injury in the previous year. All participants gave their written informed consent to participate in the study after being informed about the procedure. Leading up to the experiments, participants followed their regular training program. Also, participants did not perform any unaccustomed or intense training session or match 48h before the protocol. The study was approved by the ethics committee of Sud-Est II of Lyon. All participants performed two eexperimental testing sessions at the same time of day separated by at least 48h during the players’ regular training period (3–5p.m.), aiming to diminish the effects of residual fatigue and circadian variation. The first session included all anthropometric measurements and the isokinetic torque testing of knee muscles. In the second session, participants performed sprint running tests, and deceleration performance was characterized.

### Experimental Design

The present study used a cross-sectional design to investigate the relationship between the knee muscle isokinetic torque and explosive neuromuscular capacity in both dominant leg (DL) and non-dominant leg (NDL), and deceleration performance in female professional soccer players. The deceleration performance was assessed by radar-derived kinetics measurements, including horizontal braking force, braking power, and braking impulse. The protocol consisted of two experimental testing sessions during the competitive season. The first session included the isokinetic torque testing of knee muscles, and the second testing session was the field assessment, including the linear deceleration test.

### Experimental Sessions

#### Isokinetic Torque Evaluation

Isokinetic measures were taken on the dominant (i.e., the kicking leg, DL) and non-dominant (i.e., contralateral, NDL) legs in a random order. The participants performed a general warm-up for 10min on a cycle ergometer at a resistance of 1watt.kg^−1^ (70–80 RPM). Thereafter, participants were seated on an isokinetic dynamometer (Contrex, CMV AG, Dübendorf, Switzerland) with hips flexed at 80° (0°=full hip extension), and standard stabilization strapping was placed across the chest, pelvis, and distal thigh (Zhang et al., 2021). The axis of the dynamometer was visually aligned with the lateral femoral condyle. The range of movement was set from 100° of knee flexion (starting position) to 20° (0°=knee fully extended). The torque and angle signals were recorded at 256Hz. The gravity compensation procedure was performed according to the manufacturer’s instructions. Raw torque-time curves were extracted from the original instantaneous torque dataset. And then, raw data were filtered by second low-pass 20Hz aim to reduce baseline noise ranges. The data were processed in MATLAB (MathWorks, version 2018b, Natick, MA, United States). The torque threshold was set at 1% of the maximal peak torque for each angular velocity to determine the onset and offset of muscle contraction (Zhang et al., 2021). Before the isokinetic testing, all participants completed a familiarization of isokinetic contraction consisting of KE and KF submaximal contractions in concentric mode at 60° and 240°.s^−1^, and in eccentric mode at 30°.s^−1^. After a 5min recovery period, the participants performed three maximal knee extension-flexion concentric contraction tests (60°.s^−1^, 240°.s^−1^) and eccentric contraction tests (30°.s^−1^) in a random order. The recovery period between each repetition and each trial was 30-s and 3-min, respectively. In the entire testing session, each participant was orally encouraged to give their maximal effort with a command to push (concentric)/pull (eccentric) as fast and hard as possible against the isokinetic dynamometer arm.

#### Maximal Horizontal Deceleration Test

All testing procedures were completed on the same third-generation artificial turf surface with rubber granules, specifically for outdoor field-sport events with standard meteorological conditions (temperature: 9°–14°; wind: <2-m/s; and degree of humidity: <52%). Each participant was asked to wear the same sprigged training shoes and team training attire as usual. All the participants performed a similar 20-min on-field dynamic warm-up protocol specific to soccer (e.g., dynamic stretching, lunges, and squat jump). Participants then performed three progressive 30-m sprints and 10-m of progressive deceleration phase representing a subjective moderate, intense, and quasi-maximal effort. Participants also performed three submaximal decelerations after a 20-m sprint run. After 4min of passive rest, subjects performed three maximal sprints of 20-m from a crouching position (staggered stance) finished by the fastest stop possible, interspersed by a 4-min passive recovery period.

**Figure 1:**
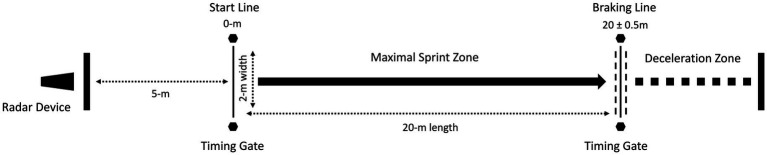
The linear deceleration assessment design.

A Stalker radar device (Stalker ATS II, Applied Concepts, Dallas, TX, United States, 46.9Hz) was attached to a heavy-duty tripod positioned 5-m behind the starting line at a height of 0.9-m above the ground (corresponding approximately to subject’s center of mass) which aimed to record the raw velocity-time curve during the maximum deceleration tests. Furthermore, the 20-m sprint times were recorded using timing gates (TC Brower Timing System, Draper, United States) set to a height of 90-cm. Times were recorded to the nearest 0.01s. Each sprint started from a stationary split stance position with the front foot positioned 30-cm behind the timing gate to prevent a false trigger. Participants were instructed to initiate their start with no backward step or “rocking motion” and sprint as fast as possible. Each participant performed two trials of maximal sprints interspersed by a passive recovery period of at least 4-min. The participants performed three maximal deceleration tests (Figure 1). During the maximal deceleration tests, subjects were instructed to stop immediately as quickly as possible after 20-m sprint running (e.g., braking line) and then backpedal to the 20-m line (Figure 1). Any 20-m time that was 5% slower than the best 20-m split time achieved during the sprint test was disregarded for analysis.

### Measurements and Data Analysis

#### Peak Torque and H/Q Ratio

The best PT value across the three maximal repetitions at 240°, 60°, and −30°.s^−1^ was used for final analysis. Conventional H/Q ratios were calculated by dividing KF concentric PT by KE concentric PT at 60°.s^−1^ (H_con60_/Q_con60_) and 240°.s^−1^ (H_con240_/Q_con240_). A functional H/Q ratio was calculated by dividing KF eccentric PT at 30°.s^−1^ by KE concentric PT at 60°.s^−1^ (H_ecc30_/Q_con60_) and 240°.s^−1^ (H_ecc30_/Q_con240_), respectively.

#### RTD and RTD H/Q Ratio

The absolute RTD was calculated as the slope of the torque-time curve between 0 and 100 ms interval (i.e., ∆torque/∆time) after the contraction onset for KE and KF at 60° and −30°.s^−1^. RTD H/Q ratios were calculated by dividing the KF RTD with the KE RTD both for concentric (RTD H_con100_/Q_con100_) and eccentric (RTD H_con100_/Q_ecc100_) contractions.

#### Maximal Deceleration Mechanics

All data were collected using the software Stalker Acceleration Testing System (STATS; v5.0.2.1, Applied Concepts, Dallas, TX, United States) provided by the radar device’s manufacturer. A custom-made data analysis routine (MATLAB, R2018b, Natick, MA, United States) computed the kinetic variable including the horizontal braking force (*F_H_*), power (*P_H_*), and impulse (*I_H_*) calculated from the start to the end of the maximal sprint deceleration as previously reported (Harper et al., 2020). The instantaneous horizontal acceleration (*a_H_*) was calculated by the gradient of the time-velocity curve as:


(1) $$a_{H}\left(t\right)=\frac{\partial v}{\partial t}$$

And the net horizontal force *F_H_*(*t*) during the deceleration period was then modeled over time:


(2) $$F_{H}\left(t\right)=m.a_{H}\left(t\right)+F_{aero}$$

where *a_H_*(t) is the acceleration at time *t* and *m* is the body mass. In addition, ∂t is the variation in time, and ∂v is the variation in velocity. *F_aero_* is the air friction, which was calculated with the equation *F_aero_* as the aerodynamic friction force to overcome during sprint running computed from sprint velocity and an estimated body frontal area and drag coefficient (Arsac and Locatelli, 2002).


(3) $$F_{aero}\left(t\right)=\left(0.2025*Height^{0.725}*Mass^{0.425}\right)*0.266$$

Besides, the horizontal power (*P*_H_(*t*)) was calculated with the following equation:


(4) $$P_{H}\left(t\right)=F_{H}\left(t\right)*V_{H}\left(t\right)$$

Instantaneous horizontal impulse [*I_H_*(*t*)] was calculated between each data point during the deceleration phase using the change in the momentum.


(5) $$I_{H}\left(t\right)=\partial v*mass$$

**Figure 2:**
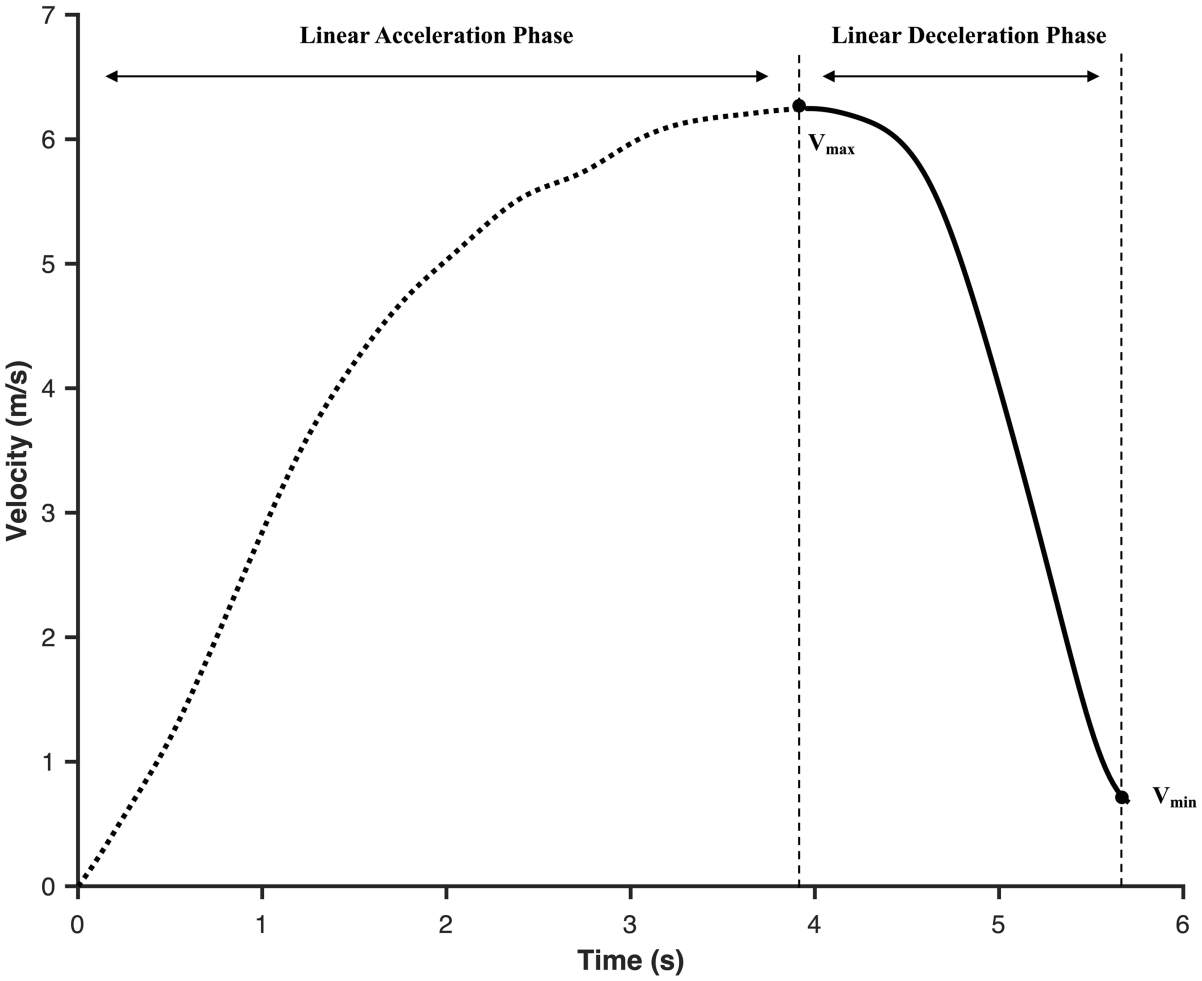
Example of velocity-time profile showing the acceleration and the deceleration phase. *V*_max_, maximum velocity defining start of deceleration phase; *V*_min_, lowest velocity defining end of deceleration phase.

The average horizontal braking force (*F_ave_*), braking power (*P_ave_*), and braking impulse (*I_ave_*) were calculated using the average of all instantaneous *F_H_*, *P_H_*, and *I_H_*, during the entire deceleration phase. Furthermore, the maximum braking force (*F_max_*), power (*P_max_*), and impulse (*I_max_*) were obtained as the highest value of all instantaneous *F_H_*, *P_H_*, and *I_H_* values during the entire deceleration phase (Figure 2).

### Statistical Analysis

Before performing the statistical analysis, the Shapiro–Wilk test was used to assess the data’s distribution normality. Pearson’s product-moment correlation coefficients (*r*) were calculated to examine the relationship between deceleration variables and isokinetic test parameters. The magnitude of the correlation coefficient was interpreted using criteria: very weak (0.11–0.19), weak (0.20–0.39), moderate (0.40–0.59), strong (0.60–0.79), and very strong (0.80–1.00). The coefficient of determination (*r*^2^) was used to indicate the shared variance of correlation and presented as a % (*r*^2^*100). The value of *p* was set at 0.05 significance level. All statistical procedures were performed with R software (R 3.5.0, R Core Team, Vienna, Austria).

## Results

The peak torque, RTD and H/Q ratio results are shown in Tables 1–3. Kinematic and kinetic variables of the sprint and deceleration test are presented in Table 4. The significant correlation coefficients between the kinematic variables of maximal deceleration and lower limb torque profiles are presented in Table 5.

**Table 1:** Peak torque (PT in N.m) of knee extensor (KE) and knee flexor (KF) in dominant leg (DL) and non-dominant leg (NDL).

Angular velocity	Muscle	DL		NDL	
		Mean	95% CI	Mean	95% CI
240°.s^−1^	KE	89.89±20.23	[78.21–101.56]	91.46±21.48	[79.06–103.87]
	KF	55.23±16.08	[45.95–64.51]	54.92±15.79	[45.80–64.04]
60°.s^−1^	KE	129.56±21.08	[117.39–141.73]	130.02±19.49	[118.76–141.27]
	KF	72.04±14.00	[63.96–80.12]	73.59±17.09	[63.72–83.46]
−30°.s^−1^	KE	160.65±34.61	[140.66–180.63]	163.49±32.55	[144.69–182.28]
	KF	97.81±11.42	[91.35–104.28]	101.19±14.19	[92.82–109.56]

All data are presented as mean±standard deviation with 95% confidence interval.

**Table 2:** Rate of torque development (RTD in N.m.s^−1^) of knee extensor (KE) and knee flexor (KF) in dominant leg (DL) and non-dominant (NDL).

Velocity	Muscle	DL		NDL	
		Mean	95% CI	Mean	95% CI
60°.s^−1^	KE	612.15±139.11	[531.83–692.48]	658.23±124.91	[528.37–788.09]
	KF	485.50±91.02	[437.82–533.17]	488.01±93.75	[438.90–537.11]
−30°.s^−1^	KE	233.75±58.85	[202.92–264.57]	203.95±47.95	[178.83–229.06]
	KF	231.17±49.01	[205.86–256.86]	238.64±56.43	[209.08–268.20]

All data are presented as mean±standard deviation with 95% confidence interval.

**Table 3:** Traditional H/Q ratio (H/Q) and KF RTD to KE RTD ratio (RTD H/Q) in dominant leg (DL) and non-dominant leg (NDL).

Variable	DL		NDL	
	Mean±SD	95% CI	Mean±SD	95% CI
Conventional H/Q				
H_con60_/Q_con60_	0.56±0.14	[0.49–0.64]	0.56±0.08	[0.51–0.61]
H_con240_/Q_con240_	0.61±0.10	[0.55–0.67]	0.60±0.09	[0.54–0.65]
Functional H/Q				
H_ecc30_/Q_con60_	0.75±0.16	[0.66–0.84]	0.75±0.08	[0.70–0.80]
H_ecc30_/Q_con240_	0.76±0.07	[0.72–0.80]	1.13±0.17	[1.03–1.23]
RTD H/Q				
RTD H_con_/Q_con_	0.81±0.24	[0.67–0.95]	0.74±0.15	[0.66–0.83]
RTD H_con_/Q_ecc_	2.07±0.67	[1.67–2.46]	2.45±0.94	[1.90–2.99]

Con, concentric contraction; Ecc, eccentric contraction; 240, 240°.s^−1^; 60, 60°.s^−1^; 30, −30°.s^−1^. All data are presented as mean±standard deviation with 95% confidence interval.

**Table 4:** Kinematic and kinetic variables during the sprint and maximum deceleration.

	Variable	Mean±SD	95% CI
Sprint	Velocity at 20m (m.s^−1^)	6.60±0.37	[6.41–6.79]
	20m split time (s)	3.87±0.12	[3.81–3.94]
Deceleration	Approach velocity (m.s^−1^)	6.41±0.59	[6.13–6.69]
	*F_ave_* (N)	300.38±57.20	[273.31–327.46]
	*P_ave_* (W)	1,007.24±207.33	[909.11–1,105.37]
	*I_ave_* (N.s^−1^)	6.00±1.24	[5.41–6.58]
	*F_max_* (N)	444.18±119.97	[387.40–500.96]
	*P_max_* (W)	1,694.72±360.72	[523.99–1,865.45]
	*I_max_* (N.s^−1^)	12.70±2.60	[11.47–13.94]

*F_ave_*, average braking force; *P_ave_*, average braking power; *I_ave_*, average braking impulse; *F_max_*, maximum braking force; *P_max_*, maximum braking power; *I_max_*, maximum braking impulse. All data are presented as mean±standard deviation with 95% confidence interval.

**Table 5:** Pearson’s significant correlation results between the deceleration ability variable and lower limb peak torque, RTD H/Q ratio in DL and NDL.

	Variable	Correlation coefficient, *r* [95% CI]	Coefficient of determination % (*r*^2^)	Qualitative inference	*p*
*F_ave_*	PT_60__KE_Con_NDL	−0.54 [−0.83 to −0.02]	30	Moderate	0.044
	PT_30__KE_Ecc_NDL	−0.71 [−0.90 to −0.30]	51	Strong	0.004
	RTD_KE_Ecc_DL	−0.54 [−0.83 to −0.03]	29	Moderate	0.040
*P_ave_*	PT_30__KE_Ecc_NDL	−0.70 [−0.90 to −0.27]	49	Strong	0.005
	RTD_KE_Ecc_DL	−0.63 [−0.87 to −0.16]	39	Strong	0.015
*I_ave_*	PT_60__KE_Con_NDL	−0.55 [−0.84 to −0.02]	30	Moderate	0.043
	PT_30__KE_Ecc_NDL	−0.68 [−0.89 to −0.23]	46	Strong	0.008
	RTD_KE_Ecc_DL	−0.54 [−0.85 to −0.01]	29	Moderate	0.044
*F_max_*	PT_30__KE_Ecc_NDL	−0.61 [−0.86 to −0.12]	38	Strong	0.020
*P_max_*	PT_240__KE_Con_DL	−0.57 [−0.85 to −0.06]	33	Moderate	0.033
	PT_240__KE_Con_NDL	−0.58 [−0.85 to −0.07]	33	Moderate	0.031
	PT_240__KF_Con_DL	−0.58 [−0.85 to −0.07]	34	Moderate	0.030
	RTD H_con_/Q_con__DL	−0.61 [−0.86 to −0.11]	37	Strong	0.022
	RTD H_con_/Q_con__NDL	−0.59 [−0.85 to −0.08]	34	Moderate	0.028
*I_max_*	PT_30__KE_Ecc_NDL	−0.75 [−0.92 to −0.37]	56	Strong	0.002
	RTD H_con_/Q_con__DL	−0.57 [−0.84 to −0.05]	32	Moderate	0.035

*F_ave_*, average horizontal braking force; *P_ave_*, average braking power; *I_ave_*, average braking impulse; *F_max_*, maximum braking; *P_max_*, maximum force power; *I_max_*, maximum impulse; PT, peak torque; KE, knee extensors; KF, knee flexors; Con, concentric contraction; Ecc, eccentric contraction; 240, 240°.s^−1^; 60, 60°.s^−1^; 30, −30°.s^−1^.

### Relationship Between Peak Torque, RTD, and Deceleration Performance

Strong correlation was found between KE PT at −30°.s^−1^ in NDL and *F_ave_* (*r*=−0.71, *p*=0.044), *P_ave_* (*r*=−0.70, *p*=0.005), *I_ave_* (*r*=−0.68, *p*=0.008), *F_max_* (*r*=−0.61, *p*=0.02), and *I_max_* (*r*=−0.75, *p*=0.002), respectively, accounting for 51, 49, 46, 38, and 56% of the explained variance (Table 5). In addition, KE PT at 60°.s^−1^ in the NDL leg was moderate correlated with *F_ave_* (*r* =−0.54, *p*=0.044) and *I_ave_* (*r*=−0.55, *p*=0.043) accounting for 29 and 30% of the explained variance, respectively (Table 5). Furthermore, moderate correlations were found between *P_max_* and KE PT at 240°.s^−1^ in DL (*r*=−0.57, *p*=0.033), and NDL (*r*=−0.58, *p*=0.031), accounting for 33 and 34% of the explained variance, respectively. Besides, there was a moderate correlation between KF PT at 240°.s^−1^ in the DL and *P_max_* (*r*=−0.58, *p*=0.03), accounting for 34%, of the variance (Table 5). Moreover, a moderate/strong relationship was found between RTD of KE at −30°.s^−1^ in DL and *F_ave_* (*r*=−0.54, *p*=0.04), *I_ave_* (*r*=−0.54, *p*=0.044), and *P_ave_* (*r*=−0.63, *p*=0.015), accounting for 30, 29, and 39% of the explained variance, respectively (Table 5).

### Relationship Between Strength Ratios and Deceleration Performance

Interestingly, strong correlations between *P_max_* and RTD H/Q at 60°.s^−1^ in DL (*r*=−0.61, *p*=0.022), and moderate correlation between RTD H/Q in NDL and *P_max_* (*r*=−0.59, *p*=0.028) were observed, accounting for 37 and 34% of the variance, respectively (Table 5). In the DL, a moderate correlation was observed between RTD H/Q at 60°.s^−1^ and *I_max_* (*r*=−0.57, *p*=0.035), accounting for 32% of the variance (Table 5). In contrast, no significant relationship was observed between conventional H/Q ratio, functional H/Q ratio and deceleration performance (all *p*>0.05).

## Discussion

The present study is the first to investigate the relationships between the capacities of the knee muscles’ rapid torque production and mechanical variables related to deceleration performance in female professional soccer players. The main finding indicated a moderate to strong correlation of the knee extensors’ eccentric maximal isokinetic torque and early phase RTD with the horizontal braking force, power, and impulse (all *r*<−0.54). In addition, a moderate correlation was observed between RTD H/Q and horizontal braking force, power and impulse (*r*<−0.57).

Numerous previous studies already demonstrated that a high level of lower limb eccentric strength promotes deceleration performance and change-of-direction performance (Lockie et al., 2012; Spiteri et al., 2013; Jones et al., 2017; Harper et al., 2021). This finding was in agreement with Jones et al. (2017), who indicated the female soccer player with higher eccentric strength of the quadriceps at 60°.s^−1^ related to the horizontal GRF at penultimate contact during the 180° COD task (Jones et al., 2017). As aforementioned, the higher knee extensor muscles could contribute to efficient KE eccentric force output to absorb the impact force by the support and reduce the mechanical stress on joint structures when the trunk is oriented backward (Hewit et al., 2011; Sasaki et al., 2011), thus outputting a higher *P_max_*, *I_max_* to rapidly decelerate. This finding was in agreement with Jones et al. (2017), who indicated the female soccer player with higher eccentric strength of the quadriceps at 60°.s^−1^ related to the horizontal GRF at penultimate contact during the 180° COD task (Jones et al., 2017). It, therefore, appears that current finding could partially support the recent study of Harper et al. (2021) who indicated the KE eccentric torque at 60°.s^−1^ provided significant correlation (*r*=−0.63) with deceleration time and distance to stop after 20-m sprinting (Harper et al., 2021). Thus, it appears that the eccentric KE torque at low velocity was the main predictor (37.2–50.4%) of deceleration performance due to the higher KE eccentric torque that can partly contribute to the braking *F_max_* and *I_max_* to decelerate.

Besides, KE and KF concentric torque at high angular velocity largely correlates with the *P_max_*, which confirms the influence of thigh concentric strength on deceleration performance previously reported (Harper et al., 2021). Such a correlation can be explained by the fact that power output depends on force and velocity capacities, and then braking power may be better correlated with torque production at high velocity. Furthermore, the KF concentric torque could help to maintain hip joint stability as well as dynamically control knee flexion and the whole-body position during the deceleration maneuvers, which could contribute to a better deceleration performance (Sole et al., 2008; Jones et al., 2017). In contrast, no relationship was found between KF eccentric torque and braking ability which was in line with Harper et al. (2021). Even if further studies are still needed to determine the length changes of knee flexors during the braking phase, it can be hypothesized that they are mostly contracted in an “isometric” way, which may explain the absence of correlation between KF isokinetic eccentric torque and deceleration performance.

Interestingly, a negative correlation was found between KE RTD during eccentric contraction and *F_ave_*, *P_ave_*, *I_ave_*, but no correlation between the KE RTD during concentric contraction was observed. These results indicated that a high ability to rapidly produce eccentric force during eccentric contraction following the onset of contraction plays an important role in predicting braking ability. As mentioned previously, the maximal deceleration maneuver requires higher motor control demands of the lower limb, including rapid neuromuscular recruitment with higher eccentric force output of KE, and neuromuscular efficiency. The magnitude of the rapid contractile impulse of KE represented by RTD could contribute to an effective neuromuscular activation property of KE at the initial ground contact instant (i.e., pre-heel strike phase) before the more prolonged stance phase (i.e., heel to toe-off phase; Hewit et al., 2011; Jordan et al., 2015; Maffiuletti et al., 2016; Dos’Santos et al., 2017). Thus, the higher KE RTD in eccentric contraction may contribute to a better passive torque rise (higher contribution of passive elements) to counterbalance the magnitude of GRF during maximal deceleration maneuvers resulting in higher braking force production. Yet, no relationship between KE RTD in concentric contraction and deceleration performance suggests that it may not be pertinent to measure the RTD during the concentric contraction to predict the braking force, perhaps due to the KE eccentric work during the stance phase of deceleration.

Meanwhile, whereas knee joint stability was considered as the paramount capacity to maintain a stable body center of gravity during dynamic movements such as jumping, loading, and cutting, which depend on the ability to rapidly reach a given antagonist to agonist joint moment relationship measured by the RTD H/Q (Zebis et al., 2009, 2011), a moderate correlation was found between the RTD H_con_/Q_con_ ratios and deceleration performance (e.g., *P_max_*, *I_max_*). It can be hypothesized that the ability to have optimal neuromuscular control between the KE and the KF following a higher RTD H/Q can increase the stiffness of the lower limb to efficiently and quickly absorb the GRF while maintaining knee joint dynamic stability (De Ste Croix et al., 2017). As a result, the higher RTD H_con_/Q_con_ ratio could induce better knee joint dynamic stability following the rapid knee joint moment and contribute to efficient KE eccentric force output to absorb the impact force by the support and reduce the mechanical stress on joint structures when the trunk is oriented backward (Hewit et al., 2011; Sasaki et al., 2011), thus outputting a higher *P_max_*, *I_max_*. However, the extent of no relationship between RTD H_con_/Q_ecc_ and deceleration performance remains poorly understood. In contrast, no correlation was found between the traditional H/Q ratio (i.e., conventional or functional ratios) and deceleration performance. This result is not surprising if one refers to recent articles investigating these ratios and their arguments for improving performance and preventing injury (Grygorowicz et al., 2017). Indeed, these ratios are criticized for being calculated on different angles of maximum torque production for the two muscle groups, which could detect knee force imbalance but do not predict knee muscle co-activation capacity. Thus, it seems interesting to measure the RTD H_con_/Q_con_ ratios in soccer players to determine to what extent their deceleration performance is limited or not by an RTD H/Q ratio deficit. As results, it now seems preferable to focus on angle-specific H/Q ratios and explosive force ratios (RTD H/Q ratios; Greco et al., 2012; Zhang et al., 2021).

Previous studies revealed that the symmetry between DL and NDL might play an essential role in change-of-direction performance (Thomas et al., 2020) and ACL injury (Brophy et al., 2010). Soccer is a single-leg-dominant laterality sport because the soccer player nearly always uses the DL to manipulate the ball (i.e., kicking or passing), whereas the NDL is often used to control dynamic body stability (Wong et al., 2007). Moreover, the most striking observation to emerge from the present results was that greater correlations were obtained between the peak torque of the NDL and braking ability as well as between the RTD H/Q of the DL and braking ability. Firstly, the fact that the DL RTD H/Q ratios were correlated to deceleration performance can be explained by the fact that this leg is not often used to stabilize the body in specific soccer movements. Therefore, the player’s ability to be explosive while quickly stabilizing the knee with this leg could be essential. We can assume that the DL became decisive in the ability to decelerate quickly and stop in a stable position before performing another explosive action because this is not the most common action performed by this leg. Therefore, other parameters must influence the relationship between muscle capacities of the DL and the NDL and braking ability. We can assume that the two legs have different functions in this type of movement. The NDL might cushion impacts while the other might be more involved in stabilizing the body and maintaining balance. This could explain why the PT of the DL and the RTD H/Q ratio of the DL was identically correlated to braking ability. The first one contributes to the dissipation of kinetic energy, and the second one participates in body stabilization.

### Limitation

When interpreting the current findings, two limitations should be considered. Firstly, our population consisted merely of 14 female professional football players, which might influence the strength and reliability of the drawn conclusions; thus, future studies should investigate more participants, especially add the male soccer players to confirm the present results, and make comparison between the gender. Secondly, isokinetic dynamometers are often more compliant due to the compression of soft-tissue at the beginning of the contraction and noisier than strain gauges, potentially increasing errors in the calculation of RFD (Maffiuletti et al., 2016).

## Conclusion

Taken together, the present study highlights the close relationship of KE eccentric PT and RTD, as well as RTD H/Q and deceleration performance. These findings revealed that for athletes with greater KE torque in eccentric at a slow angular velocity and concentric contractions at moderate angular velocity, the KE RTD of eccentric contraction could produce higher horizontal braking force and impulse. Furthermore, the RTD H/Q ratio in concentric contraction might determine knee joint dynamic stability allowing to consider new goals for improving performance and preventing injuries in soccer players. Moreover, it appears that training should aim to reduce the asymmetry between the two legs and, if possible, improve the explosive neuromuscular force of the NDL because this could enhance deceleration performance and reduce the potential ACL injury risk factor during deceleration and change-of-direction tasks.

### Practical Recommendation

Given the high-intensity linear deceleration demand during the competitive match and its role in determining the change of direction performance, the current findings suggest that athlete requires to develop the eccentric strength of the knee extensor to improve the deceleration performance following the braking ability increased (i.e., braking force, braking power, and braking impulse). For instance, an inertial eccentric-overload training program including flywheels device could be used to develop the eccentric strength (Petré et al., 2018) and also reduce the risk of musculoskeletal injury (i.e., ACL) associated with decelerating (Donelon et al., 2020). Besides, the present study also indicated the critical role of early RTD during the eccentric contraction in determining the deceleration performance, suggesting that resistance training with the highest possible acceleration should be performed to promote early RTD. Moreover, considering the early phase RTD H/Q ratio may more accurately reflect the potential for dynamic knee joint stabilization during rapid limb movement (Zebis et al., 2011). It was suggested that early phase H/Q RTD might help identify players at a potentially greater risk for a knee injury during the deceleration task.

## Data Availability Statement

The raw data supporting the conclusions of this article will be made available by the authors, without undue reservation.

## Ethics Statement

The studies involving human participants were reviewed and approved by “Sud-Est II” of Lyon. The patients/participants provided their written informed consent to participate in this study.

## Author Contributions

QZ, AL, DW, and CH conceived and designed the experiments and wrote the manuscript. QZ and AL performed the experiments. QZ, AF, and CH analyzed the data and contributed materials and analysis tools. All authors contributed to the article and approved the submitted version.

## Funding

The present study was funded by the China Scholarship Council (CSC; No. 201708070091).

## Conflict of Interest

The authors declare that the research was conducted in the absence of any commercial or financial relationships that could be construed as a potential conflict of interest.

## Publisher’s Note

All claims expressed in this article are solely those of the authors and do not necessarily represent those of their affiliated organizations, or those of the publisher, the editors and the reviewers. Any product that may be evaluated in this article, or claim that may be made by its manufacturer, is not guaranteed or endorsed by the publisher.
